# Extracellular Vesicles in Lung Cancer Metastasis and Their Clinical Applications

**DOI:** 10.3390/cancers13225633

**Published:** 2021-11-11

**Authors:** Michela Saviana, Giulia Romano, Patricia Le, Mario Acunzo, Patrick Nana-Sinkam

**Affiliations:** 1 Department of Internal Medicine, Division of Pulmonary Diseases and Critical Care Medicine, Virginia Commonwealth University, Richmond, VA 23298, USA; michela.saviana@vcuhealth.org (M.S.); giulia.romano@vcuhealth.org (G.R.); patricia.le@vcuhealth.org (P.L.); mario.acunzo@vcuhealth.org (M.A.); 2 Department of Molecular Medicine, University La Sapienza, 00161 Rome, Italy

**Keywords:** extracellular vesicles, metastasis, lung cancer, non-coding RNA, therapy

## Abstract

**Simple Summary:**

Cancer cells are known to interact with the surrounding extracellular environment to facilitate tumorigenic processes. One mechanism by which cancer cells communicate with each other and their environment is through extracellular vesicles. These vesicles contain various biological molecules that are secreted by parental cells for delivery to target recipient cells. Over the past several decades, knowledge of the structure and contents of extracellular vesicles has provided valuable insight into tumor biology. Considering this information, researchers have begun examining the potential of these vesicles for developing novel biomarker classifiers as well as therapeutic strategies.

**Abstract:**

Extracellular vesicles (EVs) are heterogenous membrane-encapsulated vesicles secreted by every cell into the extracellular environment. EVs carry bioactive molecules, including proteins, lipids, DNA, and different RNA forms, which can be internalized by recipient cells, thus altering their biological characteristics. Given that EVs are commonly found in most body fluids, they have been widely described as mediators of communication in several physiological and pathological processes, including cancer. Moreover, their easy detection in biofluids makes them potentially useful candidates as tumor biomarkers. In this manuscript, we review the current knowledge regarding EVs and non-coding RNAs and their role as drivers of the metastatic process in lung cancer. Furthermore, we present the most recent applications for EVs and non-coding RNAs as cancer therapeutics and their relevance as clinical biomarkers.

## 1. Introduction

Despite the advent of targeted therapeutics, immunotherapy, and strategies for early detection, the battle against cancer wages on. In the past two decades, investigators have identified small and ubiquitous particles termed extracellular vesicles (EVs) that appear to play an increasing role in the development of several diseases, including cancer. EVs are small vesicles containing cytoplasmic constituents, including proteins, lipids, and nucleic acids. Constitutively released by every cell [1,2], EVs are freely transported throughout the circulatory system, where they can interact with cells in both the micro- and macroenvironment. EVs have been detected in various biological fluids, including blood, cerebrospinal fluids, bronchoalveolar fluid, breast milk, urine, and saliva [3,4,5,6,7,8], raising their potential as clinically relevant biomarkers.

To date, investigators have demonstrated that EVs play roles in a broad scope of biological and pathological processes [1]. EV-mediated delivery of biological effectors is often essential to the maintenance of cell physiology. EVs are involved in signaling that controls immune responses, by triggering or suppressing inflammation [9,10,11,12,13,14,15,16,17]. Moreover, EVs are released in the brain in a synaptic-dependent way, and allow for neural communication [18]. Also, EVs are involved in tissue repair, coagulation, and stem cell maintenance [19,20,21].

In cancer, investigators have implicated EV biology in many malignant processes, including inflammatory responses, cell proliferation, migration, invasion, angiogenesis, epithelial-to-mesenchymal transition, and the formation of pre-metastatic niches [22,23,24].

Lung cancer remains as one of the most prevalent malignancies across the world, accounting for more deaths than any other reported malignancy [25]. For 2021, the American Cancer Society estimates that 235,000 new cases and 131,000 deaths will result from lung cancer in the United States alone [26]. This disease has two distinct histological subtypes: non-small cell lung cancer (NSCLC) (76% of lung cancer cases) and small cell lung cancer (SCLC) (13% of lung cancer cases) [27]. Tobacco smoking is linked to approximately 90% of lung cancers and contributes to the high mutational burden often observed with this malignancy [28]. Notably, there is an increasing population of never-smokers who are being diagnosed with lung cancer as well [25]. Overall, the five-year survival rates are dismal at ~20%, but the overall mortality rates have declined; this trend is likely driven by decreased incidence, improvements in screening criteria and implementation, increased sensitivity of imaging modalities, and more effective chemotherapeutics and treatment regimens (immunotherapy, targeted drugs against EGFR and ALK) [25,27,29,30]. In light of this recent progress in lung cancer screening and treatment, patients are still often diagnosed at a late stage, at which point therapies with meaningful long-term responses are limited, and even with treatment, these patients often succumb to metastatic disease [26,31,32].

Increasing our understanding of EV-mediated cell-to-cell communication and EV contents may prove valuable in elucidating additional mechanisms for lung cancer metastases and clinical biomarkers [33]. In this manuscript, we review the role of EVs in lung cancer metastasis and the implications for lung cancer therapies.

## 2. Types and Characteristics of EVs

EVs were first identified in 1946 [34] and were initially considered to be essential for physiological activities such as the cellular elimination of cytotoxic materials [35]. A few years later, their role as mediators of intercellular communication began to be studied, with the discovery that EVs harbor important bioactive molecules, such as nucleic acids [36], which can be internalized by recipient cells, consequently perturbing their biological phenotype [35].

Currently, three main types of EVs have been characterized based on differences in their size, biogenesis, and mechanism of release (see Table 1).

### 2.1. Biogenesis and Release

Exosomes: Exosome biogenesis involves the endosomal system and is driven by the endosomal sorting complex required for transport (ESCRT) [37,38]; Rab GTPases (including Rab7A, Rab11, Rab27A, Rab27B, and Rab35), ALG-2-interacting protein X (ALIX), and vacuolar protein sorting-associated protein (VPS4) are support factors contributing to ESCRT regulation and exosomes release [39]. Exosome biogenesis may also occur in an ESCRT-independent manner, in which proteins including tetraspanins are involved [40,41].

Exosome release is mediated by lipid-protein and protein-protein interactions that coordinate fusion with the plasma membrane. Among the proteins involved in this process, SNAREs and Rab GTPases are the most studied [42,43,44,45]. Exosome release is also regulated by environmental factors, including stress-related conditions; indeed, it has been reported that irradiation, hypoxia exposure, and cisplatin treatment increase exosome secretion [46,47,48,49,50].

Microvesicles: MVs originate from the direct budding of the plasma membrane. This mechanism has yet to be fully defined, but it appears to be mediated by ADP-ribosylation factor 6 (ARF6) and RHOA-dependent rearrangement of the actin cytoskeleton [51]. The redistribution of phospholipids and phosphatidylserine, as well as actin contraction, are crucial factors for MV formation [52,53,54,55].

Apoptotic bodies (ABs): ABs are a product of cell disassembly via blebbing, which occurs exclusively after programmed cell death (apoptosis) [52]. The morphological formation of ABs occurs in three main steps [56]:

1. Membrane blebbing
2. Membrane protrusion formation
3. Apoptotic bodies formation

Although our knowledge of the molecular mechanisms of blebbing remains unclear [57], the formation of ABs concludes with the detachment of vesicles that are 1–5 µm in diameter.

### 2.2. EV Cargo

The EV cargo reflects the status, or molecular fingerprint, of the cells from which they originate. Contents of EVs include DNA (double-strand DNA, single-strand DNA, mitochondrial DNA), RNA (coding RNA, non-coding RNA), proteins, and lipids.

Proteins: Proteomic studies have demonstrated that the EV protein cargo is dependent on the type of EVs from which they originate [58]. One of the biggest challenges in EV characterization is identifying specific biological markers that can distinguish them from one another. Proteins that are essential to biogenesis and release of EVs are consistently present [58,59,60,61]. For example, ESCRT and its accessory proteins (Alix, TSG101, HSC70, and HSP90β) are commonly found in exosomes [39,61,62,63,64,65], as well as glycosylated proteins and proteins of the tetraspanin family [40,66,67,68]. MVs appear to be enriched with post-translational modifications, such as palmitoylation and myristoylation, which can help drive the loading of proteins into MVs [69,70]. MVs also contain proteins associated with microtubules and cytoskeleton networks [58,71], as well as integrins, heat shock proteins, and metalloproteinases [71,72,73,74]. Investigators have also identified some transcriptional factors within MVs [75]. Unlike MVs and exosomes, ABs contain whole organelles; therefore, the presence of proteins from mitochondria (HSP60), the nucleus (histones), Golgi apparatus, and endoplasmic reticulum is expected [61].

Lipids: A variety of lipids contained in EVs share common features with the parental cell, and thus, EV lipid composition depends on the typology of the donor cell [76]. However, some peculiar findings have been reported, including the enrichment of exosomal cholesterol, sphingomyelin, glycosphingolipids, and phosphatidylserine [77]. Wubbolts et al. were the first to describe exosomes as vesicles enriched in cholesterol and sphingolipids with properties similar to raft lipids [78]. Moreover, in a study that focused on the lipidomic characterization of EVs secreted by platelets, lipidic composition varied in accordance with the dimension of the vesicles [79].

Nucleic acids: Both RNA and DNA have been detected in EVs [59,62,77]; however, DNA has been observed more frequently in large vesicles compared to exosomes [80]. Overall, RNA molecules and principally small RNAs are the predominant nucleic acids transported within EVs. The RNA species in EVs have been examined using high-throughput RNA sequencing, identifying the presence of both protein-coding RNAs (mRNA) and non-coding RNAs (ncRNAs). Most of the RNA population contained in EVs are ncRNAs, including long non-coding RNA (lncRNA), microRNA (miRNA), ribosomal RNA (rRNA), transfer RNA (tRNA), circular RNA (circRNA), small nuclear RNA (snRNA), small nucleolar RNA (snoRNA), and piwi-interacting RNA (piRNA) [81,82,83]. In general, the profile of EV RNA is reflective of the donor cell; however, some differences have been described. For instance, some lncRNAs are enriched in EVs compared to the parental cell [84], although the mechanism of their sorting in EVs is not yet understood. A microarray study directed to identify mRNA in EVs demonstrated that some mRNAs contained in exosomes are not detected within the cells from which they originate [36]. Another study reported that many miRNAs were particularly abundant in EVs, while others were preferentially expressed in the cells [85]. Comparing the sequences of these miRNAs, the authors concluded that some specific sequences of miRNAs might facilitate their loading into EVs. Among these, the sequences GGAG and C/UCCU/G contribute to miRNA sorting, and their mutations prevent the accumulation of miRNAs in EVs. This process is regulated by the heterogeneous nuclear ribonucleoprotein A2B1 (hnRNPA2B1), which recognizes and interacts with miRNAs containing EXOmotifs, thereby acting to control loading into EVs [85]. hnRNPA2B1 has also been found to be sumoylated in exosomes, which appears to be crucial for binding the miRNAs [85].

### 2.3. EV Internalization

The mechanisms for EV internalization remain unclear and require further examination. To date, two main mechanisms for the internalization of EVs have been proposed: endocytosis and fusion with the plasma membrane [59]. EV uptake requires proteins to interact with membrane receptors on target cells in order to facilitate endocytosis [86,87,88,89]. Among these, tetraspanins, integrins, proteoglycans, immunoglobulins, and lectins have been shown to be implicated in EV internalization [90].

Overall, the manner of uptake depends on the recipient cell typology, cell status, and whether or not the cells express the receptors for the EV ligand [59,91]. Moreover, the typology and membrane composition of the EV may affect their uptake.

## 3. EVs in Lung Cancer Metastasis

To disseminate to distant organs, circulating tumor cells (CTCs) require localization to the right “soil”, namely a permissive microenvironment in terms of extracellular matrix structure and immune cell presence. The “seed and soil” theory was first described by Stephen Paget during the course of analyzing post-mortem data of breast cancer when he noticed that breast cancer metastases only appeared in specific organs [92,93]. Since his initial observation, investigators have recognized that some types of cancer, including lung cancers, metastasize to preferential distant sites. For example, in lung cancer, the preferential metastatic sites are the brain, thoracic wall, bones, and liver [94,95,96,97,98,99].

A new interpretation of the “seed and soil” theory has suggested that EVs are secreted as “seeds” by cancer cells [100]. Once the EVs enter the circulation, they can reach distant organs and release factors directly into the recipient cells, modifying the gene expression and creating a permissive and immunosuppressive microenvironment, usually known as a pre-metastatic niche [101,102].

### 3.1. Biology of Metastasis

**Figure 1:**
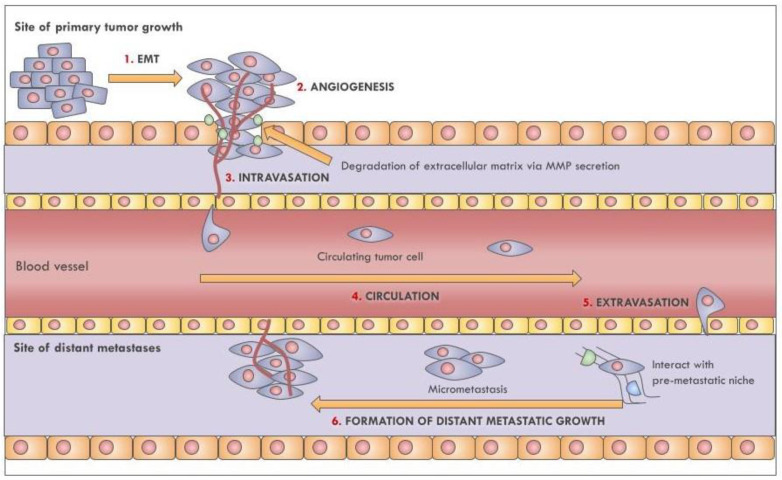
Principle phases of the metastatic process. The first step of a metastatic event involves a phenotypical transformation, specifically epithelial-to-mesenchymal transition of tumor cells (EMT); this allows for tumor invasion into surrounding connective tissue. The hypoxic conditions of the tumor microenvironment will induce tumor cells to secrete angiogenetic factors, which promote the formation of new vessels for reaching nutrients (angiogenesis), and intravasation or entry into circulation. The circulating tumor cells will travel through the turbulent circulatory system until they arrive at distant organs; here, the cells interact with pre-formed pre-metastatic niches to form new cancerous growths. Eventually, these tumors can disseminate and form further distant metastases.

Metastasis is a complex multistage process (Figure 1) that begins with the detachment of tumor cells from the extracellular matrix, followed by their colonization of the surrounding tissues; this process marks the transition from a benign nodule to an invasive malignant tumor [103]. In order to cross the basement membrane and basal lamina, cells undergo a functional and morphological change through epithelial-to-mesenchymal transition (EMT). EMT is characterized by the downregulation of key epithelial proteins, such as the E-cadherin, which are involved in epithelial cell-cell adhesion [104]. Furthermore, EMT is marked by the acquisition of actin-based membrane protrusions that promote cell migration [105]. Cancer cells also gain the ability to degrade the extracellular matrix by secreting matrix metalloproteases (MMPs); this starts the invasion process and triggers a local inflammatory response by recruiting lymphocytes, neutrophils, macrophages, and dendritic cells [103]. Following tumor growth, cancer cells form new vessels via angiogenesis to better access oxygen and nutrients from the bloodstream and to promote intravasation into the circulatory or lymphatic system (lymphangiogenesis) [106].

The survival of CTCs is not guaranteed in the circulation, nor is access to distant organs [107]. Indeed, less than 1% of circulating tumor cells (CTCs) promotes distant metastasis [103,107,108,109] due to the hostility of the bloodstream and the presence of immune cells [110].

**Figure 2:**
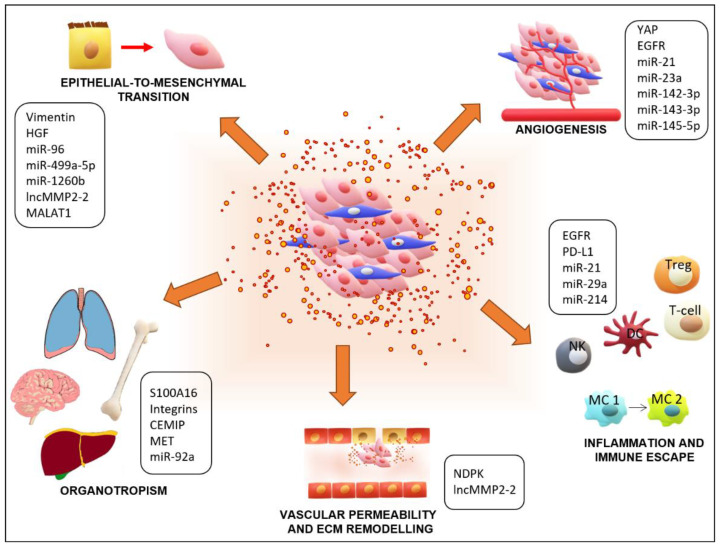
Role of extracellular vesicles (EVs) and their contents in promoting lung cancer metastasis. EVs derived from tumor cells, and other cells of the tumor microenvironment (TME), are involved in every step of the metastatic process of lung cancers. Proteins and RNAs contained in EVs can induce the first steps of metastasis, which are EMT and angiogenesis. Moreover, EVs are implicated in regulating inflammatory responses in the TME, in circulation, and at distant metastatic sites by activating T regulatory cells (Treg), altering properties of dendritic cells (DC), inhibiting the activities of T cells and natural killer cells (NK), and inducing the polarization of macrophages (MC) from type 1 to type 2. At distant sites, EVs are involved in vascular permeability and organotropism by modifying the environment of pre-metastatic niches. Notable EV content that has been shown to contribute to lung cancer metastasis is highlighted for each process.

Aggregated CTCs arrest at a distant microvascular bed and initiate the process of extravasation by secreting Angiopoietin-like 4 and metalloproteases to induce endothelial cell retraction and vascular hyperpermeability [111]. The developing micrometastasis initiates angiogenesis at the target site to allow the new tumor access to nutrients, often developing a clinically detectable large metastasis. These events require specific conditions in which EVs contribute (Figure 2). Here, we describe in detail how EVs regulate EMT, angiogenesis, immune escape, and pre-metastatic niches preparation, processes that are present in lung cancer metastasis.

### 3.2. EVs and EMT in Lung Cancer

EMT is the first step for metastatic formation, and it contributes to the acquisition of invasive abilities of cancer cells. During this process, the cancer cells undergo a phenotypic change from epithelial to mesenchymal and occasionally acquire the characteristics of cancer stem cells, a rare subpopulation of tumor cells that are able to regenerate and differentiate [112,113,114,115]. From the morphological perspective, the cuboidal epithelial cells transition into a tapered shape, causing disruption in cell-cell junctions with degradation of adhesion proteins [116,117,118]. This conversion is marked by a shift in the expression of epithelial markers (E-cadherin, Zo-1, occludin) to mesenchymal markers (vimentin, N-cadherin, fibronectin) [119]. This shift is driven by altered gene expression regulation that impedes epithelial protein production and induces mesenchymal protein production [120,121]. The change in gene expression is indispensable for cytoskeleton reorganization and subsequent cell elongation and motility. Thus, cells evolve new actin-enriched membrane protrusions that facilitate movement, including lamellipodia, filopodia, and invadopodia [117,122]. These projections assist with a proteolytic activity that acts to degrade the extracellular matrix and allows for invasion [123]. In lung cancer, numerous signaling pathways, including Wnt/β-catenin, TGFβ/Smad, IL-6/JAK/STAT3, and Notch-1, are involved in EMT [119,124,125]. These pathways lead to the activation of several transcription factors, such as SNAIL, zinc finger E-box binding homeobox 1 (ZEB1), FOXF2, and TWIST, which are responsible for the repression of endothelial markers and activation of mesenchymal genes [121,126]. The downregulation of these factors can prevent EMT. For example, miRNA-200 and the miRNA-183~96~182 cluster, which are significantly co-repressed in NSCLC, directly target FOXF2, blocking the mesenchymal phenotype [126]. Knockdown of E-cadherin has also been shown to induce a mesenchymal phenotype in A549 cells, which promotes their invasive ability through the activation of EGFR-MEK/ERK signaling. Subsequently, this pathway signaling induces the *ZEB1*-mediated increase of MMP2 expression and leads to acquired invasiveness [127].

In the last decade, researchers have started to propose a role for EVs as active attendees in the EMT process [128]. A growing body of evidence supports the notion that EVs contribute to the evolving tumor microenvironment (TME) [129,130,131]. In fact, cells secrete factors that can act as autocrine and/or paracrine signals to induce EMT and impact the TME [132,133].

A recent miRNA profiling study reported the differential expression of several oncomiRNAs in an epithelial lung cancer cell line and its induced-mesenchymal phenotype [134]. Moreover, the exosomes derived from mesenchymal cells promoted migration, invasion, and expression of mesenchymal markers in epithelial cells [134]. MiRNA-499a-5p is upregulated in both highly metastatic lung cancer cell lines and their exosomes, and this upregulation induces proliferation, EMT, and migration. In addition, treatment with exosomes derived from highly metastatic cells confers migratory and proliferative properties to recipient lung cancer cells mediated through the mTOR pathway [135].

Exosomes isolated from lung adenocarcinoma patient serum exhibit enrichment of miRNA-1260b. Furthermore, the expression of this miRNA is higher in a lung cancer cell line harboring high invasive potential (H1299) compared to a less invasive cell line (A549) [136]. Treatment with miRNA-1260b-enriched exosomes isolated from H1299 cells is sufficient to increase the invasive ability of recipient A549 cells by inhibiting sFRP1 and Smad4. Mechanistically, the inhibition of sFRP1 and Smad4 activates the Wnt/β–catenin pathway, which plays a critical role in the invasion and metastasis of lung cancer [136,137]. The Wnt/β–catenin pathway interacts with many non-coding RNA upon osteogenic differentiation [138] and the importance of this pathway in lung cancer lies in its role in causing lung cancer metastasis to bones [139].

Exosomal miRNA-96 has been reported as a lung cancer biomarker and correlates with invasive properties of different lung cell lines. Less invasive A549 cells that were treated with miRNA-96-enriched exosomes released by the highly invasive cell line H1299 exhibited improved cell viability, migration, and cisplatin resistance through inhibition of LM07 [140].

Exosomal lncRNAs have also been implicated in EMT. Lung cancer cells treated with TGFβ exhibit increased EMT properties, and co-culture experiments with these cells induced vascular permeability in human lung microvascular endothelial cells (HMVEC-L). These effects were mediated by exosomal lncMMP2-2, which induces the expression of MMP2. Furthermore, the expression of lncMMP2-2 and MMP2 in lung cancer tissue correlated with tumor progression, which suggests a potential role as a prognostic biomarker or therapeutic target [141]. Zhang et al. demonstrated that lncRNA MALAT1 was highly expressed in NSCLC patients’ serum, and its levels were associated with the tumor stage and metastasis [142]. In addition, exosomal MALAT1, derived from NSCLC patients, accelerates tumor migration and proliferation by suppressing apoptosis in lung cancer cell lines.

Protein cargo within exosomes, including vimentin, derived from a highly metastatic lung cancer cell line, was shown to drive EMT and induce proliferation, invasion, and migration in normal HBEC cells. Notably, similar results were obtained by treating HBECs with exosomes derived from the serum of late-stage lung cancer patients [143]. Another study showed that EVs released by highly metastatic cell lines contained an elevated level of HGF, and treatment with these EVs was shown to induce proliferation, invasion, and migration in low metastatic cells through the HGF/c-Met pathway [144].

### 3.3. EVs and Angiogenesis

Carcinoma cells require proximity to blood vessels in order to reach oxygen and nutrients directly. Without an adequate blood supply, tumor development and metastatic spread are hampered, with the tumor destined to become necrotic or apoptotic [145,146]. Angiogenesis is triggered by factors released by tumor cells during a phase of high growth [106,147]. Increased distance between the cells and the capillaries leads to a hypoxic state. To escape hypoxia-induced apoptosis, hypoxia-inducible factor 1α (HIF-1α) is upregulated in the TME [148,149]. HIF-1α is translocated into the nucleus, where it induces the expression of VEGF [150,151].

Endothelial cells express the VEGF receptor (VEGFR), which once activated by the ligand, initiates a transduction cascade, leading to the production of matrix metalloproteases (MMP) [152,153]. Disruption of the matrix allows for more space for endothelial cells to divide and organize into a mature network of new vessels.

Numerous studies have reinforced the importance of the EV cargo as a tumor signaling factor, which assists in creating the appropriate microenvironment and further promoting angiogenesis [154,155,156]. Among the EV cargo, numerous proteins have been reported to be involved in angiogenesis. Among these proteins, high levels of yes-kinase-associated protein (YAP) have been found in non-small cell lung cancer (NSCLC) tissues compared to normal tissue [157], and its overexpression is associated with a poor prognosis [158]. The YAP contribution to proliferation and stem cell phenotype preservation has already been discovered, and it has also been described as a pro-angiogenetic factor in different types of tumors [159,160]. Recently, the EV-mediated transfer of YAP from lung adenocarcinoma was shown to promote angiogenesis in human umbilical vein endothelial cells (HUVECs) [157].

Further evidence of the involvement of the EV cargo in angiogenesis is based on the presence of EGFR in tumor-derived MVs. EGFR is commonly overexpressed during tumor vascularization [161,162,163]. Lung cancer cells have been shown to express and secrete EGFR through MVs. MV-mediated transport of activated EGFR not only allows for incorporation of the receptor into recipient endothelial cells but can also activate the MAPK and Akt pathways, inducing the expression of VEGF [164].

The Garnis group demonstrated that the tumor suppressors miRNA-143-3p and miRNA-145-5p [165,166] were selectively packaged into EVs by lung adenocarcinoma cells [167]. EV-mediated transfer of miRNA-143-3p and miRNA-145-5p promoted tube formation by endothelial cells by targeting CAMK1D [167], an anti-angiogenic kinase. In a separate study, the same group showed that EV-encapsulated miRNA-142-3p was secreted by lung adenocarcinoma cells and transferred to endothelial cells, and promoted angiogenesis through inhibition of TGFβR1 [168]. Exosomal lncRNAs are also involved in angiogenesis. Growth arrest-specific 5 (GAS5) inhibited HUVECs’ proliferation and tube formation by increasing their apoptosis. Also, GAS5 was decreased in human lung cancer tissues, lung cancer cells, and in cell line-derived exosomes [169].

Under hypoxic conditions, investigators have demonstrated that miRNA-23a is released from lung cancer cells’ EVs to induce angiogenic pathways in vitro and in vivo [170]. Transfer of miRNA-23a within EVs enhanced the proliferation and migration of HUVECs, two necessary steps for the angiogenic process. miRNA-23a is known to be oncogenic and is upregulated in several types of cancers and in hypoxic conditions [171,172]. MiRNA-23a targets the 3′UTR of PTEN mRNA [173], the protein of which interferes with the PI3K/AKT pathway, leading to the suppression of vascular formation [173,174]. PTEN downregulation is often associated with the upregulation of VEGFR2 in glioblastoma, underlying its role in angiogenesis suppression [175].

Exposure to cigarette smoke extract (CSE) has been shown to induce human bronchial epithelial cell (HBEC) transformation [176], with an increase in STAT3 and miRNA-21 expression compared to no CSE exposure. STAT3 is already known for its effect in EMT, in CSE exposed HBEC; its knockdown led to a decrease in miRNA-21 levels in EVs [177]. Moreover, miRNA-21 was efficiently transferred through exosome-mediated communication from CSE-transformed HBE to HUVEC cells, which increases their angiogenic properties by activating VEGF. The importance of this discovery depends on the evidence that miRNA-21 was elevated in the serum of NSCLC patients and that the serum levels correlated with the smoke exposure [177]. Overall, this provides a new perspective for its employment as a potential therapeutic biomarker [178,179].

### 3.4. EVs and Immune Escape

Tumor cells express distinct antigens that are recognized by dendritic cells and lymphocytes, thereby leading to their destruction [94]. Tumor cells can promote apoptosis of T cells and natural killer cells (NK) through the Fas/FasL and PD-1/PD-L1 pathways. Alternatively, they can release EVs that promote the formation of an immune-suppressive microenvironment [180]. Indeed, tumor-derived EVs can alter immune surveillance cells through functional activation, inhibition, or polarization of immune cells [180].

Functional activation refers to the expansion and activation of regulatory T (Treg) cells and myeloid-derived suppressor cells (MDSCs), which have immunosuppressive activities in TME, and further support self-tolerance by inhibiting T cell function [180,181]. Numerous studies have reported that tumor-released EVs induce the expansion and activity of these cells [182,183].

It has been shown that exosomes from lung cancer tissues are enriched in EGFR, and that transfer of exosomes alters the properties of recipient dendritic cells (DCs), inducing tolerance and activation of Treg cells [184]. Yin et al. reported elevated expression of miRNA-214 in the tissue and plasma of patients with various cancers, including lung cancer. Tumor-secreted miR-214 can be delivered through MVs, leading to the expansion of Tregs by targeting PTEN in CD4^+^ T cells. Moreover, miRNA-214 promotes the secretion of IL-10 in Tregs, which encourages tumor growth in vivo [185].

Functional inhibition targets the silencing of normal antitumor responses that are mediated by DC, NK cells, and T-lymphocytes. CD8+ cytotoxic lymphocytes target tumor cells, and their dysfunction has been reported in many types of cancers [186,187,188]. The inhibition of CD8+ cells is mediated by the activation of checkpoint pathways such as PD-1/PD-L1. Cancer cell surface expression of PD-L1 interacts with PD-1 on T cells, leading to their inhibition [189]. EVs may harbor and transfer PD-L1 to many cell types, including tumor cells, macrophages, and DC, thus contributing to the formation of an immunosuppressive microenvironment [180] EVs containing PD-L1 have been detected in the serum of patients with cancer [190,191,192]. Specifically, Li et al. found that the level of exosomal PD-L1 in NSCLC patients correlated with the tumor stage, size, number of positive lymph nodes, and presence of distant metastasis [192]. EVs secreted by tumor cells also harbor immunoinhibitory factors, such as Fas ligand and TNF-related apoptosis-inducing ligand, constraining the effect of antitumor immune cells and promoting their apoptosis [193,194].

The polarization of immune cells involves resident macrophages in the TME. M1-type macrophages are proinflammatory, secreting interleukin-12 (IL-12), and promote the apoptosis of tumor cells. M2 macrophages secrete the anti-inflammatory interleukin-10 (IL-10), which supports tumor progression [195]. Tumor-associated macrophages mainly show an M2 phenotype, possibly resulting from the polarization of M1 macrophages to M2 macrophages [195,196,197].

Tumor-derived exosomes can deliver functional tyrosine kinase receptors and activate the MAPK pathway in monocytes, leading to the inhibition of apoptosis-related caspases [198]. In order to drive the formation of metastases, it is crucial that CTCs survive in the circulation. In the bloodstream, CTCs interact with circulating immune cells, including macrophages, NK, lymphocytes, neutrophils, Treg, monocytes, and DCs, which can then intercept and attack them before extravasation [199,200]. Mechanisms through which CTCs can escape from immune cells include the expression of PD-L1 and CD47 and the alteration of FAS/FASL, which can promote T cell apoptosis or protect tumor cells from apoptosis [199]. Ye et al. found that the number of CTCs in stage IV NSCLC patients negatively correlates with the number of NK cells and CD3+, CD4+, and CD4+/CD8+ lymphocytes, while a positive correlation was found with the number of Treg cells [201].

### 3.5. Pre-Metastatic Niches and CTCs’ Homing

As previously discussed, metastases to specific organs tend to be tumor type-specific. In order to successfully seed distal organs, CTCs require an optimal environment; thus, the site of metastasis is manipulated and adapted to promote the attachment, survival, and outgrowth of CTCs [202].

The remodeling of pre-metastatic niches is a step-by-step process that includes: modifications in vascular permeability through upregulation of metalloproteases, angiogenesis, extracellular matrix remodeling, and alterations in immune cell accumulation [100,182,203,204]. Many factors contribute to this process, including those released from tumor cells and the recruitment of hematopoietic precursor cells and mesenchymal stem cells that allow for the engraftment of CTCs and their growth [205,206]. Pro-angiogenetic factors, such as VEGF-A and proinflammatory cytokines, including TNFα and TGFβ, are released by cancer cells [205,207,208,209]. Cancer-associated fibroblasts modify the extracellular matrix by secreting metalloproteases and promoting the proliferation and invasion of tumor cells [210,211]. Hematopoietic precursor cells have been shown to express VEGFR and can colonize the pre-metastatic lung sites before the CTCs arrive [212,213]. In many tumor types, exosomes derived from cancer and non-cancer cells drive the generation and support of pre-metastatic sites upon CTCs’ arrival [182,214,215,216]. It has been demonstrated that platelet-derived MVs induce proliferation and invasion in lung cancer cell lines and can stimulate the expression of MMP-9, VEGF, Il-8, and HGF in HUVEC cells [217]. Many types of cancer metastasize to the lungs after EVs have contributed to remodeling of the microenvironment. For example, nucleoside diphosphate kinase A and B (NDPK)-enriched exosomes derived from breast cancer cells promote the migration of pulmonary vascular endothelial cells, improving their permeability [218]. In human renal carcinoma, MVs that are shed from a subpopulation of CD105+ cells induce angiogenesis and pre-metastatic niche formation. Treatment with these MVs enhanced lung metastases in immunocompromised severe combined immunodeficient (SCID) mice [219].

An interesting study by Xu et al. showed that human brain microvascular endothelial cells (HBMECs) promoted survival and apoptosis resistance in SCLC cell lines under stress conditions by releasing factors that modulate mitochondrial activity [220]. Elevated levels of S100A16, a member of the S100 family and a partner of the annexin family of proteins, were associated with brain metastasis. Co-culture with HBMECs led to the upregulation of S100A16 in SCLC cells, and this effect was suppressed by treatment with an inhibitor of exosome release, suggesting that HBMEC derived S100A16 contributes to SCLC metastasis to the brain. In a pivotal study, Peinado and colleagues elegantly demonstrated the effect of melanoma-derived exosomes on metastatic niche preparation. Labeled exosomes derived from highly metastatic B16-F10 cells were injected in naïve mice. Exosomes were then detected in specific target organs, including the lung, where they induced endothelial permeability and expression of genes related to extracellular matrix remodeling and inflammation (including heat-shock proteins, S100a8, and S100a9). In addition, a high number of bone marrow-derived cells were present at metastatic sites, and exosomal transfer of MET from melanoma cells to bone marrow progenitor cells promoted the metastatic effects [23].

In lung cancer, exosomal miRNA-21 and miRNA-29a bind and activate TLR8 and TLR7 in recipient immune cells, inducing the secretion of pro-metastatic cytokines through the activation of NF-kB [221]. In 2016, Liu and colleagues demonstrated that cancer-derived exosomes, enriched in small nuclear RNAs, activated TLR3 in lung epithelial cells, which induced the secretion of chemokines and promoted the formation of a pre-metastatic niche by recruiting neutrophils in the lung. Furthermore, they found a correlation between the level of TLR3 in adjacent tissue to lung cancer with the presence of neutrophils. Additionally, a reduction in overall survival was observed in lung cancer patients with high levels of both TLR3 and neutrophil infiltration [222].

Another critical study showed that the pattern of integrins contained in tumor-derived exosomes is implicated in organotropism and is responsible for organ-specific exosome uptake. Moreover, exosomal integrins can affect the expression of pro-metastatic genes through the activation of Src [223]. Further, the tetraspanin-8 is significantly enriched in EVs released by metastatic non-small cell lung cancer (NSCLC) cells when compared to a non-metastatic cell line. NSCLC cell lines that were treated with EVs overexpressing tetraspanin-8 displayed increased invasiveness [224]. Since the tetraspanin-integrin complex is essential for tumor-derived exosome recruitment at the pre-metastatic site, their involvement in the reprogramming of cells at these pre-metastatic niches can be speculated [225].

EVs containing miRNA-92a secreted by bone-marrow-derived cells can contribute to the establishment of a liver pre-metastatic niche in a lung cancer-bearing mice model [226]. Both bone-marrow-derived EVs and miRNA-92a increased hepatic stellate cell activation and expression of collagen type I, leading to the deposition of the extracellular matrix and inducing liver metastasis in vivo. Furthermore, elevated levels of miRNA-92a were found in lung cancer patients’ serum [226].

The brain is a common site for metastasis in lung cancer, driving a poor prognosis and high mortality [227]. The brain is a hostile microenvironment for CTCs. However, tumor cell-derived exosomes may remodel this environment to support metastatic outgrowth, as recently demonstrated by Rodrigues et al. [228]. Analyses of the metastatic brain tumor led researchers to identify increased levels of cell migration-inducing and hyaluronan-binding protein (CEMIP) in the tumor compared to the surrounding stroma. Furthermore, the levels of CEMIP in primary tumors correlated with the presence of brain metastasis. Patients with high levels of CEMIP present in their brain metastasis had a poorer survival compared to patients with low levels. The uptake of CEMIP-enriched exosomes by brain endothelial cells and microglia led to an increase in proinflammatory cytokines, promoting metastasis. Interestingly, exosomes enriched in the CEMIP protein were also found in early-stage NSCLC primary tumors, with variable levels between patients, suggesting that it can serve as a potential biomarker for future brain metastasis [228].

These findings illustrate the importance of circulating EVs in pre-metastatic niche formation. Consequently, their use as biomarkers for early-stage cancer detection could reveal early disease progression. Therefore, to prevent metastasis formation, their potential as therapeutic targets should be urgently examined.

## 4. Clinical Application of EVs

Due to their unique physical characteristics (specific targeting, small size, and their ability to cross biological barriers), EVs have been increasingly examined in medical research, particularly in diagnosis and therapy, within the past several decades.

### 4.1. Biomarkers in Lung Cancer

Despite the advent of targeted therapies, immunotherapy, advances in minimally invasive surgery, and supportive care, the prognosis for lung cancer remains poor, with a five-year survival of 19% [26]. Biomarkers that can assist clinicians with an early diagnosis are essential to improving prognosis. Furthermore, clinically informative biomarkers may assist in predicting therapeutic responses. In the past several years, EVs and their contents have emerged as a potential source for developing novel non-invasive biomarkers. Most EV components (membrane proteins, lipids, and cargo nucleic acids) have been investigated as biomarkers. Hurwitz et al. demonstrated differentially expressed proteins in EVs extracted from 60 different cancer cell lines (NCI-60 that includes nine lung cell lines), indicating that EV contents have cancer specificity [229]. A study of 49 exosomal membrane proteins in 276 patients reported that NY-ESO-1 might be a reliable prognostic biomarker in NSCLC [230]. Furthermore, exosomal EGFR expression levels were found to be elevated in five out of nine cancer cases compared to healthy controls. In contrast, in the same patients, the soluble EGFR levels in plasma were distinguishable between the cancer and control patients [230]. In 2018, Wang et al. analyzed lipopolysaccharide-binding proteins (LBP) in the exosomal membrane and determined that it could distinguish between metastatic and non-metastatic NSCLC [231]. Additionally, CD171, CD151, and tetraspanin-8 (Tspan8) could also be differentiated for lung cancer patients, both NSCLC and SCLC, compared to controls [232]. As previously described, Liu et al. found an upregulation of Tspan8 in metastatic cell line-derived EVs. They also found an elevated expression of Tspan8 in serum EVs of individuals with stage III NSCLC tumors and reduced distant metastasis-free survival [224].

RNAs within EVs are also potential cancer biomarkers [233]. Exosomal nucleic acids (exoNAs) from NSCLC patient plasma for common *BRAF*, *KRAS*, and *EGFR* mutations showed higher sensitivity for assessing clinical outcomes as compared to plasma ctDNA [234]. Hur et al. analyzed EV-derived plasma DNA plasma and bronchoalveolar lavage fluid (BALF) from NSCLC patients. They discovered higher concordance with a conventional tissue biopsy compared to circulating free DNA (cfDNA). In addition, the EGFR p.T790M mutation was detectable in patients developing EGFR-TKI resistance [235].

Numerous studies have described cargo ncRNAs as biomarkers in lung cancer [236] (Table 2).

In particular, miRNAs including miRNA-21 [241,243,260], miRNA-23b-3p, miR-10b-5p [249,261], miRNA-139-5p miRNA-200b-5p, miRNA-378a, miRNA-379, and miRNA-4257 were found to be dysregulated in EVs of lung cancer patients compared to healthy controls [237,241,249,261,262]. Circulating miRNAs can be used as predictive (e.g., miRNA-21, miRNA-122, and miRNA-205), diagnostic (e.g., miRNA-21, miRNA-126, and miRNA-205), and prognostic (e.g., miRNA-21, miRNA-16, and let-7) biomarkers for lung cancer [250]. A recent meta-analysis suggested that EV-derived lncRNA MALAT1 could be a promising biomarker for NSCLC screening; however, due to its low specificity, further validation is required [263]. Zang et al. describe a positive correlation of MALAT-1 levels in serum-derived exosomes with the tumor stage and lymphatic metastasis [142]. LncRNA growth arrest-specific transcript 5 (GAS5) was downregulated in patients with NSCLC. This lncRNA was inversely expressed to the tumor stage [264]. In 2019, Rao et al. showed that exosome lncRNA HAGLR and CTCs could serve as biomarkers in NSCLC patients [256].

EV lncRNAs have also been implicated in drug resistance. For example, in NSCLC, lncRNA RP11-838N2.4 was linked to erlotinib resistance [258], while lncRNA H19 was linked to gefitinib resistance [259].

Recently, investigators have examined post-transcriptional RNA modifications (reviewed in [265,266,267]) as diagnostic biomarkers. In a recent paper, Nigita et al. identified first-time differences in the editing level of mature miRNAs in circulating exosomes of NSCLC patients [268,269].

Promising biomarkers have also been reported in other body fluids. In urine, leucine-rich alpha-2-glycoprotein 1 (LRG1) was upregulated in tissue and urinary exosomes from NSCLC patients [270]. Sun et al. profiled salivary EVs in healthy and lung cancer patients and described four proteins (BPIFA1, CRNN, MUC5B, and IQGAP) as potential biomarker candidates in lung cancer [271]. miRNA-1-3p, miRNA-144-5p, and miRNA-150-5p were also identified in pleural lavage [257]. A comparison between EVs from pleural fluid of lung cancer, pulmonary tuberculosis, or pneumonia patients demonstrated differential miRNA expression patterns [272].

Indeed, the combination of more than one biomarker increases the accuracy. For example, combining EVs RNA and ctDNA increased the sensitivity of EGFR mutation detection in NSCLC patients’ plasma [273]. Despite these exciting advances, the translation of exosome-based technologies to clinical application still requires standardized methods for isolating EVs and must take into consideration the heterogeneity of cancer-derived EVs and their immunological effects [182,274,275,276].

### 4.2. Therapeutic Targets

Given the crucial role of EVs in cancer progression and the metastatic process, many studies have focused on the use of EVs as therapeutic targets through inhibition of EV formation in tumor donation cells, inhibition of EV uptake in recipient cells, and the ablation of circulating EVs.

Key molecules in EV biogenesis and secretion are considered potential candidates for EV-targeting treatment and include neutral sphingomyelinase 2 (nSMase2) and the Rab family (Rab22, Rab7a, Rab27a, Rab27b, Rab11, etc.). nSMase2 is known to regulate EVs’ production, and its targeting can reduce tumor growth and distant metastasis [155,277]. This protein regulates EV-associated miR-210 secretion and promotes angiogenesis, therefore, affecting the capacity for metastasis in breast cancer [155]. Yokoi et al. used an ovarian cancer orthotopic mouse model derived from an *nSMase2* KD (knockdown) cell line and observed a significant reduction in metastases to the peritoneal cavity [278]. Fabbri et al. used GW4869, a nSMase2 inhibitor, and observed a decrease in pulmonary metastasis in mice with lung cancer [221]. Inhibition of nSMase in PC-3 prostate cancer cells did not affect EV release, suggesting that EV biogenesis regulation may be cancer-specific [279].

Targeting Rab proteins is another strategy to downregulate EV release in cancer. In lung cancer cells (A549), EV release was significantly downregulated following targeted inhibition of Rab27a and Rab32 [280,281]. Inhibition of Rab27a led to a reduction in primary tumor growth and the number of lung metastases in a 4T1 cell (metastatic cell line) xenograft but not in a TS/A (non-metastatic) xenograft model, indicating a possible therapeutic use of Rab27 inhibition for reducing metastases [282]. The inhibition of Rab37 reduced lung cancer stemness in vitro and in vivo [283].

The use of dimethyl amiloride (DMA), a Ca2+ blocker used in cardiology, has shown an inhibitory effect on EV release in vitro (colon, lung, and lymphoma cell lines) [284] and in vivo [285]. The in vivo studies indicated that DMA enhances the antitumor efficacy of cyclophosphamide, suggesting it should be considered as part of combination therapy for cancer [285]. In glioblastoma, heparin (a heparan sulfate inhibitor) inhibited EV uptake and downregulated cell migration [88]. Dynasore, a clathrin-mediated endocytosis pathway inhibitor, caused a significant decrease in EV uptake in lymphoma [286] and normal endothelial cells [287].

In PC9 cells, a NSCLC cell line, EVs extracted following gefitinib treatment reduced cisplatin’s antitumor effect, which is associated with an increase in autophagy. The authors demonstrate an antagonistic effect of gefitinib and cisplatin due to gefinitib-stimulated EVs. They suggested a washout period to completely eliminate TKI-related exosomes before the next cycle of chemotherapy [288].

Targeting EV release/uptake machinery has two main challenges: (1) EV release/uptake pathways remain poorly understood, and (2) There is a lack of methods for selective EV inhibition. Since EVs are involved in normal physiological communication, therapies should be as tumor-specific as possible to minimize disruption to the natural homeostatic function of EVs. Improvement in understanding and targeting tumor-selected pathways, as well as tumor-specific markers on the EV surface and EV cargo, will be essential to successful targeting.

### 4.3. EVs as Vehicles for Therapy

EVs have been considered an ideal vehicle for therapeutic agent delivery (drug/nucleic acid) [269]. In particular, their stability in physiological and pathological conditions and decreased immunogenicity compared to other nanocarriers are two attractive features. EVs can selectively deliver their cargo based on the presence of specific membrane proteins [289]. In 2015, Hoshino et al. describe an association between exosomal integrins and preferential tissue metastasis: the presence of α_6_β_4_ and α_6_β_1_ was associated with lung metastases, while exosomal integrin α_v_β_5_ was linked to liver metastases [223]. α2,3-linked sialic acid on exosomes is linked to spleen-specific delivery [290]. CD63^+^ EVs target neuronal and glial cells. Furthermore, CD63^−^ selectively targets dendritic cells [291], while the Tspan8/integrin a4 complex selectively targets EVs to pancreatic cells [292].

Before using EVs as a delivery system, it is crucial to evaluate the EV biodistribution, lifetime, circulation kinetics, and overall pharmacokinetics. EVs accumulate preferentially in the liver, spleen, kidney, and gastrointestinal system [293]. EV biodistribution is determined by the targeting, cell source, and route of administration [294,295]. Wiklander et al. reported that EV membrane proteins are essential for biodistribution [295]. This is supported by evidence showing that proteinase K treatment of EVs prior to intravenous injection in mice significantly reduced lung distribution. Interestingly, proteinase K treatment did not affect macrophage uptake [296]. CD47 is a “don’t eat me” signal present on the membrane of many tumor cells and EVs; thus, it can protect cells and EVs from phagocytosis by monocytes and macrophages [297,298,299]. Studies on the structure, functions, and uses of CD47-inhibition assess the potential and challenges of the CD47-SIRPα signaling pathway in anticancer therapy [300]. Engineered EVs for therapy can utilize CD47 to increase the lifetime of EVs [298]. Furthermore, by using a function-blocking CD47 antibody, it is possible to modulate multiple EV-mediated signals between breast carcinoma cells and endothelial cells that are important for supporting tumor growth and metastasis [301]. On the other hand, using exosome-SIRPa, which is the ligand of CD47, is sufficient to increase the tumor cell phagocytosis and prime an antitumor T cell response [302]. Another method to improve the circulation time and biodistribution of EVs is by coating the EVs with polyethylene-glycol (PEG) conjugated with anti-EGFR nanobodies or with streptavidin [303,304].

Different types of cells are under investigation as potential sources of EVs as vehicles for drug delivery. Dendritic cells (DCs), as APCs, were used as cancer immunotherapy, but their use in clinical practice has bene found to be challenging [305,306]. Dendritic cell-derived exosomes (DEXs) have been studied as an alternative to DCs as anticancer vaccines, given their ability to activate the immune response. A few clinical trials, both phase I and phase II, demonstrate that DEXs can initiate the adaptative and innate immune system, encouraging the future use of this strategy for cancer treatment, including for NSCLC, melanoma, and colorectal cancer [307]. Currently, a phase II clinical trial is evaluating the use of antigen-loaded dendritic cell-derived exosomes as a vaccine in NSCLC (http://clinicaltrials.gov NCT01159288 (accessed on 1 November 2021). Furthermore, many in vitro and in vivo studies demonstrate that DEX can also serve as an effective drug delivery system [307,308,309,310,311]. Mesenchymal stem cells (MSCs) possess anti-inflammatory and strong regenerative effects [312]. They have therapeutic potential against various diseases, such as cardiovascular disease, liver injury, renal injury, and neural injury [313]. Furthermore, MSC-derived exosomes (MEVs) largely contribute to MSCs’ therapeutic effects [314,315]. There is an ongoing investigation seeking to improve MEV-based drug delivery systems for clinical use in different diseases, including cancer [316,317,318,319].

Macrophage-derived EVs have also been considered for immunotherapy, given their ability to activate the T cell response and act as a drug delivery system [310]. Aminoethyl anisamidepolyethylene glycol (AA-PEG) exosomes that are loaded with paclitaxel accumulated in high concentrations in cancer cells and improved therapeutic outcomes on lung metastases [320]. EVs from tumor cells (TEVs), and in particular, autologous TEVs, have been used for therapeutic purposes. In a recent paper, Guo and colleagues evaluated the therapeutic potential of TEVs in the context of malignant pleural effusion (MPE) [321]. The authors obtained robust results when assessing the safety, immunogenicity, and clinical activity of autologous TEVs-patching methotrexate [321]. Recently, a TEVs-based platform for anti-miR-21 delivery and magnetic resonance imaging (MRI) has been investigated in breast cancer [322]. The study demonstrated that TEV-mediated anti-miR-21 delivery reduced doxorubicin (DOX) resistance in breast cancer cell lines; the authors also demonstrated a tumor-specific accumulation of TEV using MRI. This paves the way for the use of TEVs for future applications in cancer molecular imaging and therapy [322]. Finally, in the last five years, milk and red blood cells (RBCs) have been considered a good source of EVs for therapy due to their abundance and cost-effective benefits [310].

One of the primary challenges of EV-mediated delivery is the lack of target specificity. The “perfect” carrier must be stable but also target cell or organ type-specific. Lysosome-associated membrane protein 2 (Lamp2b) is one of the most used anchor proteins for coupling target peptides or antibodies to enhance EV targeting [323]. Lamp2b anchored with rabies viral protein (RGV), a protein that binds acetylcholine receptors, can selectively target neurons and microglia [324]; if anchored with muscle-specific peptide, they can also selectively target C2C12 muscle cells [324]. Lamp2b with cardiomyocyte-specific peptide (CMP) or cardiac targeting peptide (CTP) can result in increased EV uptake by cardiomyocytes in vivo [325,326].

EVs expressing membrane glycosylphosphatidylinositol (GPI) fused with anti-EGFR nanobodies target tumor cells with even more specificity [327]. Also, EVs with platelet-derived growth factor receptors fused to the GE11 peptide (EGFR specific) on their surface can specifically target EGFR-expressing xenografts in breast cancer [328]. Nucleic acid aptamers that can recognize specific target molecules are used to increase the specificity of targeting the PSMA aptamer in prostate cancer xenografts, EGFR aptamer in breast cancer xenografts, and survivin in colorectal cancer xenografts [329]. In another study, aptamers against nucleolin were used to target breast cancer cells that express an upregulation of this membrane protein. This resulted in an inhibition of tumor growth in vivo [330]. Qi and colleagues successfully used transferrin-conjugated super-magnetic nanoparticles to control and target murine tumors using external magnets [331].

Lastly, it is essential to consider the EV cargo and how the cargo can be packaged within vesicles. There are two approaches for EV loading: exogenous (i.e., after EV isolation) and endogenous (i.e., during EV biogenesis). Often, the best strategy is dependent on the source of the EVs and the cargo to load (reviewed in [35,332,333]).

The primary EV cargos used for therapy are nucleic acid and drugs. There are many preclinical studies and a few clinical trials that demonstrate the efficiency of EVs in many diseases. EVs that selectively deliver miRNA or siRNA to enhance the drug sensitivity, or use EVs to selectively direct drugs in tumor cells, have been of particular interest.

TRAIL-expressing EVs derived from MSCs induced apoptosis in 11 resistant and sensitive cancer lines (including three lung cancer lines, four malignant pleural mesothelioma lines, two renal cancer lines, one human breast adenocarcinoma line, and one neuroblastoma line) in a dose-dependent manner while showing no cytotoxicity in primary human bronchial epithelial cells [334].

MSC EVs overexpressing miR-122 were used to downregulate domain-containing protein 10 (ADAM10), insulin-like growth factor 1 receptor (IGF1R), and cyclin G1 (CCNG1), three proteins implicated in hepatocellular carcinoma. This downregulation enhanced the therapeutic effects of TKI sorafenib in a preclinical hepatocellular carcinoma ectopic tumor model [335]. Mendt and colleagues demonstrated that engineered EVs with siRNA or shRNA molecules targeting KRASG12D induced apoptosis of pancreatic cancer cells and increased the survival of mice harboring advanced KPC689 pancreatic tumor cells [336]. These engineered EVs (iExosome) are in an ongoing clinical trial (NCT03608631).

There remain a few concerns for the future of EVs in cancer therapy. One of the most relevant issues is the lack of standardized techniques for the isolation and purification of exosomes. In fact, one of the most active current fields of research on EVs is developing an efficient and precise method of exosome isolation [337]. Secondly, it is essential to determine the EV source. For example, tumor cell-derived EVs can contain oncogenic markers that may contribute to cancer progression. Furthermore, there is limited drug loading efficiency, which can be due to limited space (EVs contain their parent cell line contents) [338]. The ongoing search for new methods to increase drug-drug loading and EVs’ yield is ongoing. Yang et al. were able to increase by up to 50-fold the exosome numbers and by more than 103-fold the exosomal cargo. This was accomplished using cellular-nanoporation [339]. Until recently, there has been no method for sufficient clinical-grade production [310]. Before EVs can be considered a reliable therapeutic platform, we must develop an efficient, reproducible, and cost-effective production method on a large scale.

## 5. Conclusions

The contributions of EVs to understanding the metastatic processes at the diagnostic and therapeutic levels have become increasingly evident in a short period. Although the role of EVs in the hallmarks of cancer continues to evolve, it is important to remain cognizant of the inherent limitations that remain associated with isolating EVs and characterizing their contents. EVs are involved in several important processes that lead to the establishment of distant metastases, making them suitable targets for cancer therapies. However, challenges with targeting cancer-specific EVs can be attributed to our limited knowledge when it comes to targeting EVs that are uniquely released by cancer cells. Fortunately, the characteristics that define EVs as excellent carriers can be utilized to create vesicles for targeted drug molecule delivery. Furthermore, the diversity and uniqueness of EV content provide researchers with valuable sources from which to discover new cancer-specific biomarkers.

## Figures and Tables

**Table 1:** Main types and characteristics of extracellular vesicles.

Variable	Exosomes	Microvesicles	Apoptotic Bodies
Size (nm)	50–150	100–2000	1000–4000
Biogenesis	Endosomal pathway	Budding of plasma membrane	Programmed cell death and blebbing of plasma membrane
Proteins	Tetraspanins, ALIX, TSG101, ESCRT, heat shock proteins	Integrins, selectins, cytoskeletal and cytosolic proteins, glycosylated and phosphorylated proteins	Histones, annexin V
Lipids	Cholesterol, sphingomyelin, ceramide, lipid rafts, phosphatidylserine	Phosphatidylserine, cholesterol, diacylglycerol, lipid rafts	Phosphatidylserine
Nucleic acids	mRNA, non-coding RNA	mRNA, non-coding RNA	DNA, RNA
Markers	CD63, TSG101, Alix, tetraspanins, Rab5a/b, HSP70, HSP9	Integrin, selectin, flittilin-2	Histones, DNA, Annexin V

**Table 2:** Principal dysregulated non-coding RNAs and their clinical applications in lung cancer.

Function	Non-Coding RNA	Clinical Applications	Reference
EMT	miRNA-200 family	Predictive, diagnostic	[126,237,238,239]
	miRNA-183~96~182 cluster	Diagnostic	[126,240]
	miRNA-499a-5p		[135]
	miRNA-1260b	Biomarker	[136]
	miRNA-96	Biomarker	[140]
	lncMMP2-2	Prognostic	[141]
	MALAT1	Prognostic	[142]
Angiogenesis	miRNA 143-3p	Biomarker	[167]
	miRNA 145-5p	Biomarker	[167]
	miRNA 142-3p		[168]
	GAS5	Biomarker	[169]
	miRNA-23a	Biomarker of disease	[171]
Immune escape	miRNA-214	Biomarker	[185]
	miRNA-21	Predictive, diagnostic, prognostic	[221,236,241,242,243,244,245,246,247,248]
	miR-29a		[221]
	miRNA-92a	Biomarker	[226]
Biomarker	miRNA-10b-5p	Prognostic, survival	[236]
	miRNA-23b-3p	Prognostic, diagnostic, survival	[236,249]
	miRNA-139-5p	Screening	[237]
	miRNA-378a	Screening	[237]
	miRNA-379	Screening	[237]
	miRNA-4257	Predictive	[241]
	miRNA-122	Predictive, therapeutic	[250,251]
	miRNA-205	Predictive, diagnostic	[250,252]
	miRNA-126	Diagnostic	[250,253]
	miRNA-16	Prognostic	[250,254]
	Let-7	Prognostic	[250,255]
	HAGLR	Prognostic	[256]
	miRNA-1-3p	Diagnostic	[257]
	miRNA-144-5p	Diagnostic	[257]
	miRNA-150-5p	Diagnostic	[257]
Drug resistance	lncRNA RP11-838N2.4	Erlotinib resistance	[258]
	lncRNA H19	Gefitinib resistance	[259]
